# Fabrication and Application of Ag@SiO_2_/Au Core–Shell SERS Composite in Detecting Cu^2+^ in Water Environment

**DOI:** 10.3390/molecules29071503

**Published:** 2024-03-27

**Authors:** Meizhen Zhang, Lin Meng, Kelgenbaev Kalyinur, Siyuan Dong, Xinyi Chang, Qian Yu, Rui Wang, Bo Pang, Xianming Kong

**Affiliations:** 1 School of Petrochemical Engineering, Liaoning Petrochemical University, Fushun 113001, China; zmz121_q@sina.cn (M.Z.); 13841348968@sohu.com (L.M.); yuan-love@outlook.com (S.D.); cxy111888@sina.com (X.C.); qyu@lnpu.edu.cn (Q.Y.); xmkong@lnpu.edu.cn (X.K.); 2 International Education College, Liaoning Petrochemical University, Fushun 113001, China; kalyinur1919@gmail.com; 3 Department of Materials and Environmental Chemistry, Stockholm University, 10691 Stockholm, Sweden

**Keywords:** core–shell, Ag@SiO_2_/Au, SERS, copper ions, detection

## Abstract

A sensitive and simple method for detecting Cu^2+^ in the water source was proposed by using surface-enhanced Raman scattering spectroscopy (SERS) based on the Ag@SiO_2_/Au core–shell composite. The Ag@SiO_2_ SERS tag was synthesized by a simple approach, in which Ag nanoparticles were first embedded with Raman reporter PATP and next coated with a SiO_2_ shell. The Ag@SiO_2_ nanoparticles had strong stability even in a high-concentration salty solution, and there were no changes to their properties and appearance within one month. The Ag@SiO_2_/Au composite was fabricated through a controllable self-assemble process. L-cysteine was decorated on the surface of a functionalized Ag@SiO_2_/Au composite, as the amino and carboxyl groups of it can form coordinate covalent bond with Cu^2+^, which shows that the Ag@SiO_2_/Au composite labelled with L-cysteine has excellent performance for the detection of Cu^2+^ in aqueous media. In this study, the SERS detection of Cu^2+^ was carried out using Ag@SiO_2_ nanoparticles, and the limit of detection (LOD) as low as 0.1 mg/L was achieved.

## 1. Introduction

Water resources have an extremely important impact on life in the world, providing an inexhaustible driving force for human birth, survival, and development. In recent decades, with the rapid development of economies around the world, the global population has surged, leading to a significant increase in people’s demand for water resources [1]. However, due to the lack of environmental awareness among humans, produced industrial wastewater is directly discharged into sewers or rivers nearby, resulting in varying degrees of pollution in a large amount of water resources [2]. This can cause ecosystem disorders, pollute the surrounding air, and pose a huge threat to human safety [3]. At present, the increasingly prominent environmental issues have attracted public attention.

Most of the wastewater contains pathogenic microorganisms, organic chemicals, heavy metals (Cu^2+^, Cd^2+^, Hg^2+^, Pb^2+^, etc.), and other harmful chemicals, especially Cu^2+^ ions, which have strong toxicity [4]; although it plays an important part in the ecosystem, once it accumulates excessively, its toxicity causes damage to human beings and the environment. Metal ions can not only remain in the body of aquatic organisms, but also penetrate into the soil, which affects the growth of plants and microorganisms, and can even be transferred to the human body through the food chain to endanger human health [5].

The detection of Cu^2+^ ions has attracted a lot of attention. The World Health Organization (WHO) stipulates that the content of Cu^2+^ ions in drinking water should not exceed 31.5 μM [6], and China requires the copper ion content in drinking water to be controlled below 1 mg/L, and the Cu^2+^ content in surface water should not exceed 0.1 mg/L [7]. At present, there are various methods to detect Cu^2+^ ions in water: the fluorescence method [8], colorimetry [9], atomic absorption spectroscopy [10], the electrochemical method [11] and spectrophotometry [12]. However, these methods all have certain drawbacks, such as low sensitivity, limited detection range, high detection cost, and the need for complex operational processes. Therefore, it is necessary to develop a method that can detect the concentration of metal ions in a timely and accurate manner.

Surface-enhanced Raman spectroscopy (SERS) is a simple, nondestructive, convenient, instant, and highly sensitive analytical method. The greatly enhanced signal-enabled SERS can detect an analyte with a lower concentration, even down to molecule level [13,14]. SERS could provide unique fingerprint spectrum information of the target; it is widely used in many fields such as pharmacology [15,16], food safety [17,18], environmental monitoring [19], and bioengineering [20]. The preparation of appropriate SERS substrates is an important prerequisite for application [21,22]. The research on the SERS substrate with core–shell structure has recently made progress in the field of materials science [23]. Tian and his team [24] pioneered a method of wrapping ultra-thin silica shells on the surface of Au nanoparticles (SHINERS). The diversity of silica shell materials includes controllable shell size, ensuring the stability of nuclear nanoparticles, and functionalized modification of the core–shell structure, making it an ideal SERS substrate [25]. As the metallic ions such as Cu^2+^ have no Raman signal [26], the use of the Raman labeling method in SERS detection is necessary. Shen et al. [27] used embedded Raman probe molecules (core–molecule–shell) in the core–shell SERS tag and employed it for a quantitative analyzing target molecule.

Amino acids have an amine and carboxylic acid group that can interact with metallic ions through strong electrostatic interactions and complexation [28]. Li and his colleagues [29] used cysteine-functionalized silver nanoparticles to detect Cu^2+^ and Hg^2+^ in water, in which the limits of detection were 10 pM and 1 pM, respectively. Chen et al. [30] prepared four amino acid-functionalized chelating cellulose particles that were effective in removing Cd^2+^ and Pb^2+^. Other measures of detecting Cu^2+^ by using various SERS biosensors are also compared in this paper (Table 1).

**Scheme 1:**
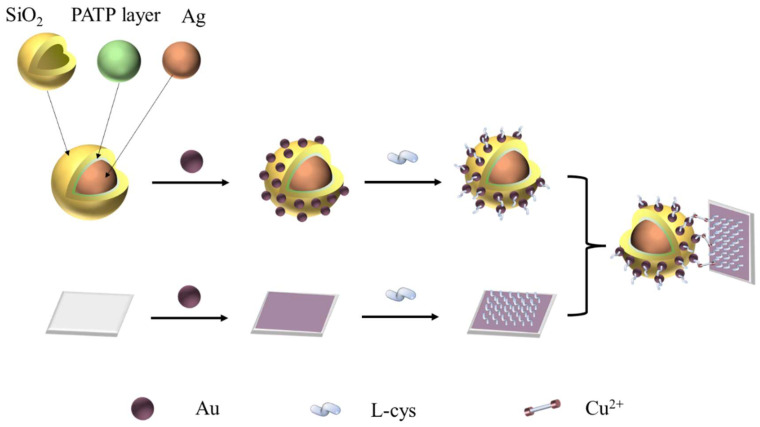
Illustration of the synthesis and application of Ag@SiO_2_-Au composite.

Herein, we successfully prepared the core–shell Ag@SiO_2_ SERS tags in which the PATP was employed as the internal standard probe molecule. And the Au NPs were modified on the surface of the core–shell structural SERS tag to form the Ag@SiO_2_/Au nanocomposite; then, L-cysteine was functionalized on the surface of the Au. L-cysteine was modified on the surface of the Ag@SiO_2_/Au nanocomposite and gold-capped glass slides via the sulfhydryl group. When Cu^2+^ presented in the system, the Ag@SiO_2_/Au nanocomposite was crosslinked onto the surface of wafers through the coordination effect. SERS detection was conducted on the wafer in order to detect Cu^2+^ ions in water media, with a detection limit as low as 0.1 mg/L. The preparation and application process of the nanocomposite are shown in Scheme 1.

## 2. Results and Discussion

### 2.1. Characterization of Ag@SiO_2_

**Figure 1:**
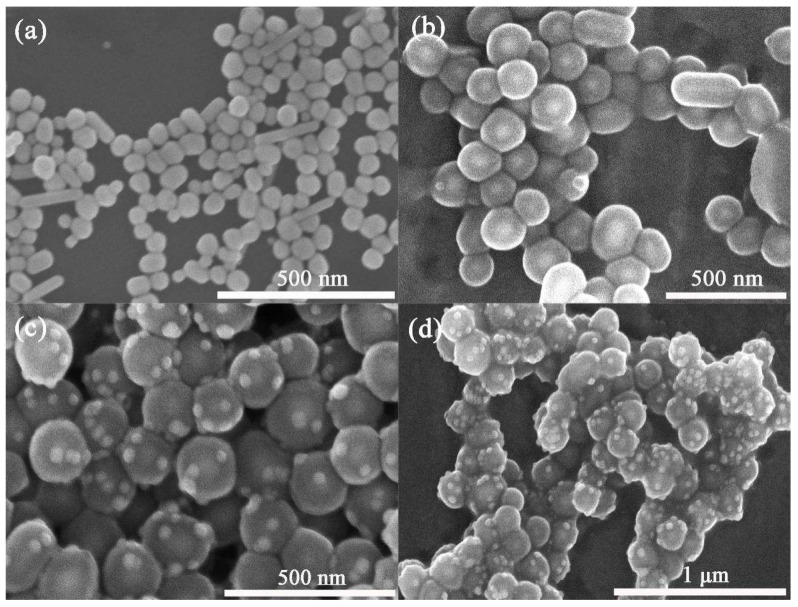
SEM images of Ag NPs (**a**), Ag@SiO_2_ tags (**b**) and Ag@SiO_2_/Au composite (**c**,**d**).

The SEM image of Ag NPs is shown in Figure 1a, in which the diameter of Ag nanoparticles ranges from 60 nm to 70 nm. The Ag@SiO_2_ SERS tags were fabricated by the Stober method which involved the addition of TEOS and NH_3_·H_2_O into the reaction system; thus, the silica seeds can grow uniformly on the surface of Ag nanoparticles. Figure 1b presents the SEM image of Ag@SiO_2_. The diameter obviously increased compared with Ag NPs as the silica shell formed. Au nanoparticles were assembled on the surface of the Ag@SiO_2_ tag; the SEM images of Ag@SiO_2_/Au composite with different magnification are shown in Figure 1c,d. The Au nanoparticles were successfully deposited onto the surface of Ag@SiO_2_ through electrostatic interaction.

**Figure 2:**
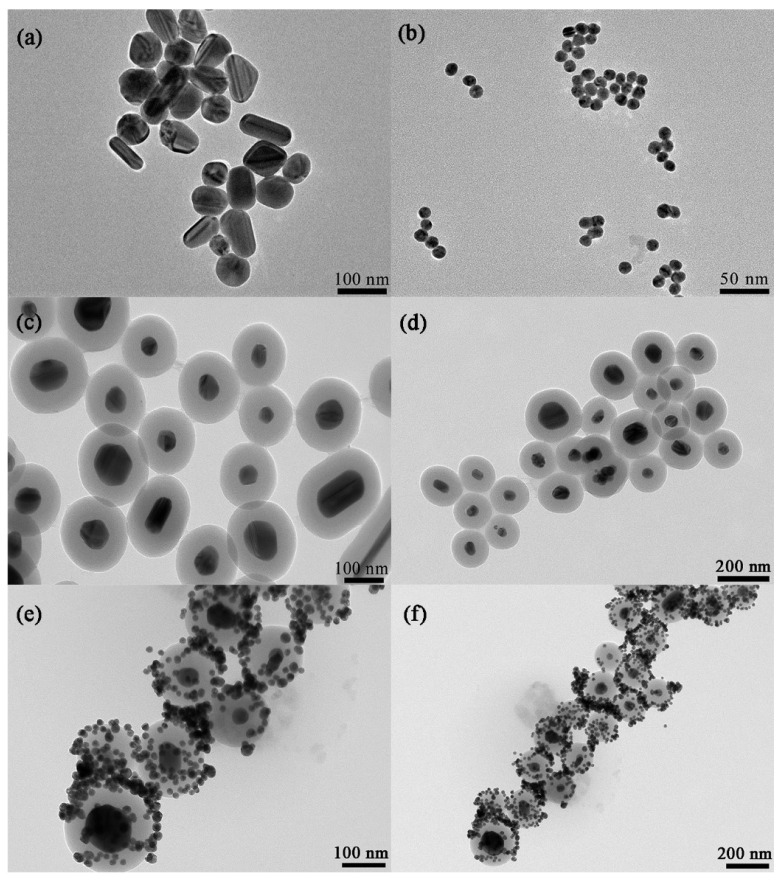
TEM images of Ag NPs (**a**), Au NPs (**b**), Ag@SiO_2_ SERS tag (**c**,**d**) and Ag@SiO_2_/Au composites (**e**,**f**).

The TEM images were used to observe the structure and morphology of the nanomaterials. In Figure 2c,d, the spherical shape of Ag@SiO_2_ nanoparticles can be observed, with the thickness of SiO_2_ of 50 nm coating Ag NPs uniformly and completely. The TEM images of the Ag@SiO_2_/Au nanocomposite are shown in Figure 2e,f, in which the Au nanoparticles are distributed on the smooth surface of Ag@SiO_2_.

The UV–vis spectra in Figure S1 present the plasmonic features of Ag@SiO_2_, Au, and Ag@SiO_2_/Au assembled with different conditions. The surface of the Au nanoparticle presents negative charge of the citrate ions which enables electrostatic self-assembly on the aminated surface of Ag@SiO_2_. Before the decoration of the Au nanoparticle, the Ag@SiO_2_ SERS tags showed a strong adsorption peak at 446 nm, and the adsorption peak of Au colloid was observed at 521 nm, which was due to the LSPR feature of metallic nanoparticles. After being anchored on the surface of Ag@SiO_2_, the adsorption spectra of Ag@SiO_2_/Au were observed, as shown in Figure S1. Two obvious peaks were observed, corresponding to the LSPR band of Ag and Au nanoparticles. The position of the LSPR band is related to the distribution and local refractive index variation of the plasmonic materials [36]. As low-concentration Au colloid was used in assembly, the adsorption peaks were located at 446 nm and 521 nm. There was nearly no change happened to the LSPR band of Ag@SiO_2_/Au compared with the Ag@SiO_2_ and Au nanoparticles. This is due to the loose distribution of Au nanoparticles on the surface of Ag@SiO_2_. With the increase in the concentration of Au colloids used in the assembling process, the red shift occurred in the LSPR band of Au nanoparticles. The adsorption peak was shifted from 521 nm to 530 nm, which was due to the dense Au nanoparticles located on the surface of Ag@SiO_2_. In addition, the adsorption intensity of Ag@SiO_2_/Au at 530 nm gradually increased with the increase in Au amount.

### 2.2. The Stability of the Ag@SiO_2_ SERS Substrate

The stability of nanomaterials is an important factor in application. The detection of metal ions is usually carried out under salty conditions, which has a significant influence on the stability of plasmonic nanoparticles. Coagulation can happen to Ag and Au nanoparticles in a high-salt solution, as the cations of inorganic salts can disrupt the double layer electric structure of metallic colloids [37,38]. The aqueous solution of NaCl (0.2 M) was chosen to evaluate the stability of Ag and Ag@SiO_2_.

**Figure 3:**
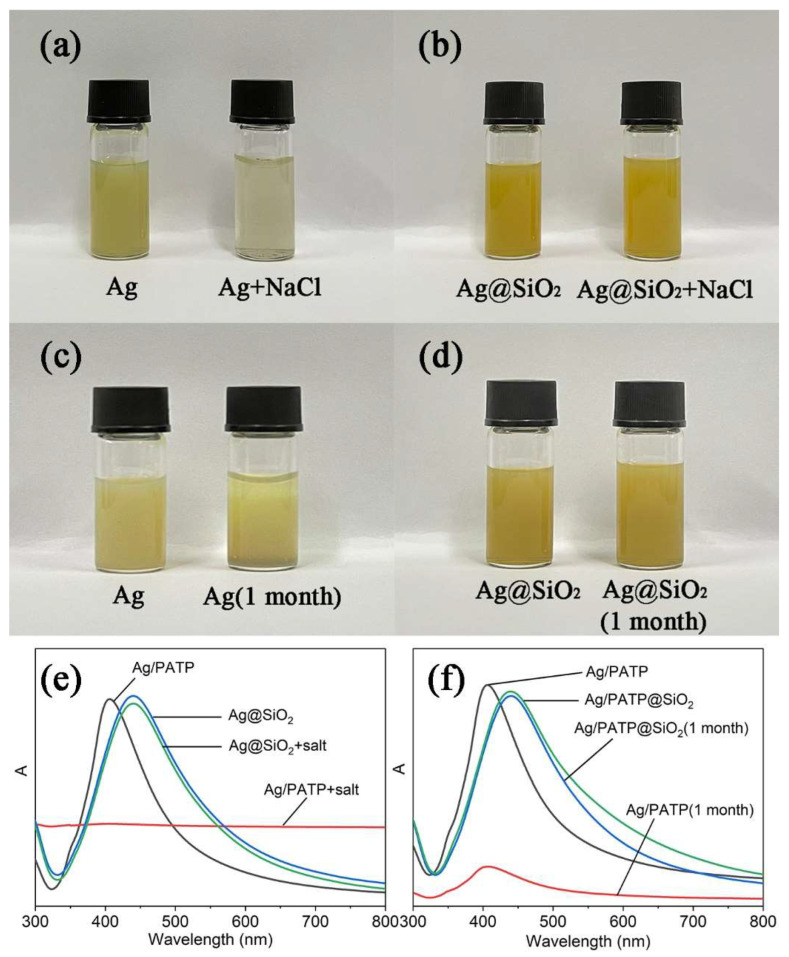
Photographic images of Ag colloids (**a**,**c**) and Ag@SiO_2_ SERS tags (**b**,**d**) under saline condition and after one month of storage. The corresponding UV–vis spectra of Ag colloids and Ag@SiO_2_ SERS tags (**e**,**f**).

The variation in the appearance of Ag and Ag@SiO_2_ colloids was directly observed regardless of the presence or absence of NaCl, as shown in Figure 3a,b. The prepared Ag and Ag@SiO_2_ colloids presented greenish–yellow appearance. After mixing with NaCl solution, the appearance of the Ag colloid almost changed to apparent, which was due to the aggregation of Ag nanoparticles. The UV–vis spectra were also employed for determining the plasmonic feature of Ag and Ag@SiO_2_ before and after mixing with the NaCl solution (Figure 3e). The adsorption strength of the Ag colloid decreased dramatically after the addition of the NaCl solution, while the adsorption spectra of Ag@SiO_2_ nearly remained unchanged after adding the NaCl solution. The stability of Ag@SiO_2_ in the salt solution assigned to the silica shell protected the Ag core.

The temporal ability is another important feature of Ag@SiO_2_ in application. Ag and Ag@SiO_2_ were stored at 4 °C in a refrigerator for one month. A partial precipitate of silver colloid was found at the bottom of the bottle (Figure 3c), which was due to the fact that Ag nanoparticles with high surface energy are prone to forming precipitation. It was noticeable that there was no significant change in the appearance of Ag@SiO_2_ (Figure 3d). The reason is that the silica shell wrapped outside had remarkable stable chemical properties, and it served as a protective layer to isolate the air circumstance. After storage for one month, the UV absorption peak of Ag colloid changed significantly, as shown in Figure 3f, and nearly no change happened to the spectra of Ag@SiO_2_. These results indicated that Ag@SiO_2_ has excellent stability.

### 2.3. Optimization of Detection Conditions

**Figure 4:**
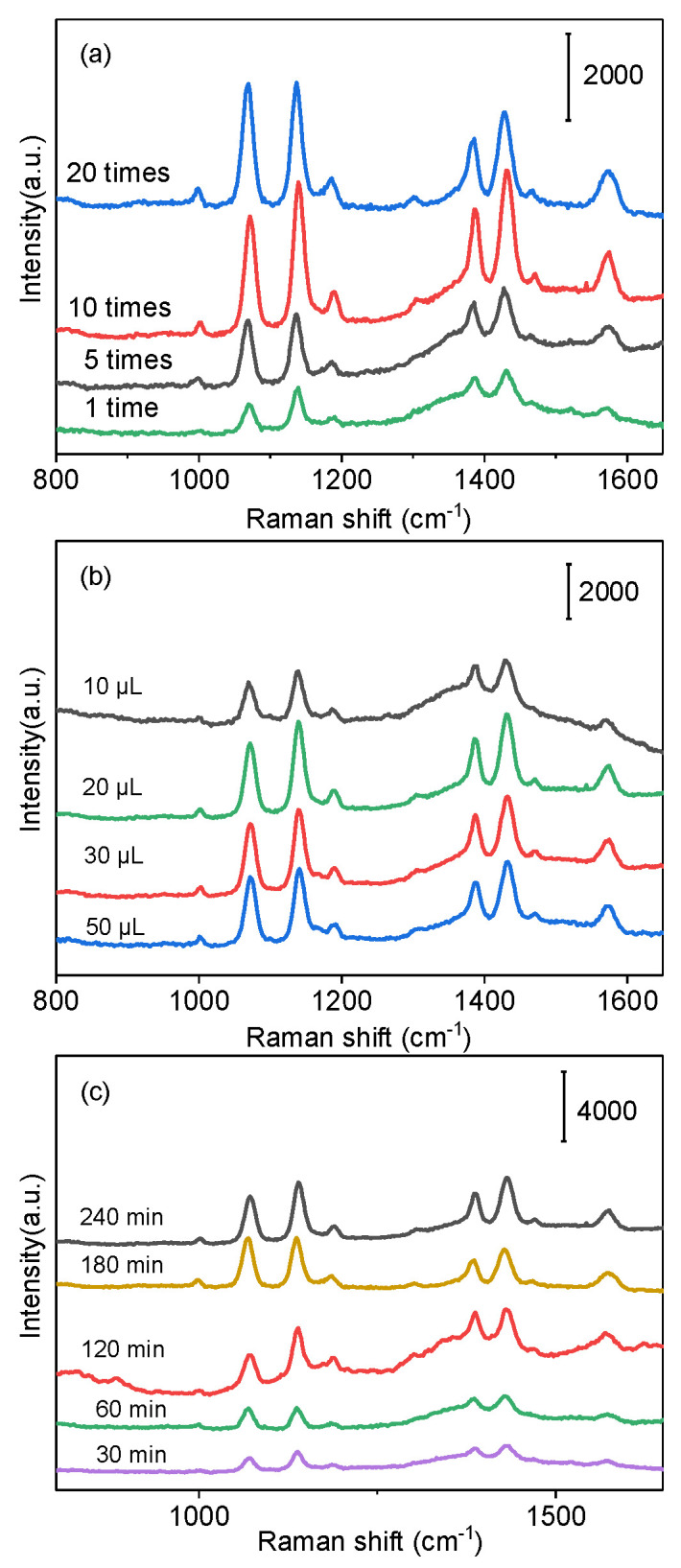
SERS spectra of L−cysteine−functionalized wafers with L−cysteine−modified Ag@SiO_2_/Au tags at (**a**) different concentrations of Au colloid used for assembled Ag@SiO_2_; (**b**) different amounts of L−cysteine used for modified Ag@SiO_2_/Au; and (**c**) different times of wafer/L−cysteine/Cu^2+^ immersing in L−cysteine−functionalized Ag@SiO_2_/Au.

The concentration of Au colloids plays an important role in the SERS performance of the sensor. The SERS spectra of PATP from Ag@SiO_2_/Au fabricated in different concentrations of Au are shown in the Figure 4a in which the volume of L-cysteine and the soaked time of wafers were set as 20 μL and 120 min, respectively. Five prominent Raman peaks at 1073 cm^−1^, 1139 cm^−1^, 1385 cm^−1^, 1433 cm^−1^, and 1586 cm^−1^ were observed, respectively, which were different from the SERS spectra of solid PATP. Three new peaks at 1139 cm^−1^, 1385 cm^−1^, and 1433 cm^−1^ did not belong to PATP; in fact, they emerged from a new substance called 4,4′-dimercaptoazobenzene (DMAB). It was the product formed by the catalytic coupling reaction on Ag nanoparticles under laser irradiation [39]. The peak at 1139 cm^−1^ was attributed to the C-H absorption vibration peak, the peaks at 1385 cm^−1^ and 1436 cm^−1^ were attributed to the stretching vibration of the N-N group and the symmetric vibration of the C-C group. The spectral band located at 1073 cm^−1^ belonged to the C-S bond vibration (a_1_ mode) [40,41]. The SERS signal strength can be affected by the concentration and reaction time of DMAB [42].

As more of Au NPs bind to the SiO_2_ shell, the corresponding amount of L-cysteine absorbed on Au NPs also increases, indirectly providing an opportunity for the interaction between L-cysteine and Cu^2+^. A low concentration of the Au colloid indicates a relatively small number of Au NPs, which means that the probability of L-cysteine binding to Cu^2+^ is greatly reduced. The weak SERS signal was obtained when original Au colloids were used. A strong Raman peak can be observed in Figure 4a as the Au colloids were concentrated 10-fold. On the contrary, higher concentration of Au nanoparticles in a certain area inhibits the generation of the SERS signal. The intensity of the SERS signal was decreased as the 20-fold-concentrated Au colloid was used for constructing Ag@SiO_2_/Au. Therefore, the 10-fold-concentrated Au colloids were selected in the subsequent experiment. Different amounts of L-cysteine (10 μL, 20 μL, 30 μL, 50 μL) were used to functionalize the surface of Ag@SiO_2_/Au. As a kind of α-amino acid, L-cysteine is composed of sulfydryl, carboxyl, and the amino group. L-cysteine could be modified on Au nanoparticles through the S-Au bond; after being immersed in a solution of the Cu^2+^ ions, the iminoacetic acid group can strongly coordinate with Cu^2+^ [43]. And the Cu^2+^-conjugated Ag@SiO_2_/Au nanoparticles can easily crosslink to the L-cysteine-functionalized substrate through the coordination interactions between L-cysteine and Cu^2+^. It can be seen that the most intense SERS signal was obtained when 20 μL L-cysteine was used in the system (Figure 4b). The Raman signal without significant enhancement was obtained with the increase in the amount of L-cysteine because the surface of Au nanoparticles was fully occupied.

The wafers modified with L-cysteine were soaked into aqueous solution of Cu^2+^ (20 μg/L) for 1 h, and the relationship between SERS intensity and soaking time of wafer/L-cysteine/Cu^2+^ in L-cysteine-functionalized Ag@SiO_2_/Au composite solution was investigated, as shown in Figure 4c. It can be observed that the SERS signal was weak when the soaking time was 30 min, and it increased gradually with the extension of soaking time. The highest intensity was obtained as the soaking time increased to 180 min, which is explained by the longer soaking time leading to the full interaction occurring between Cu^2+^ and Ag@SiO_2_/Au/L-cysteine. However, the signal intensity changed little with the soaking time was extended to 240 min. Therefore, the optimum soaking time of 180 min was chosen for detection.

### 2.4. Quantitative and Selective Detection of Cu^2+^

**Figure 5:**
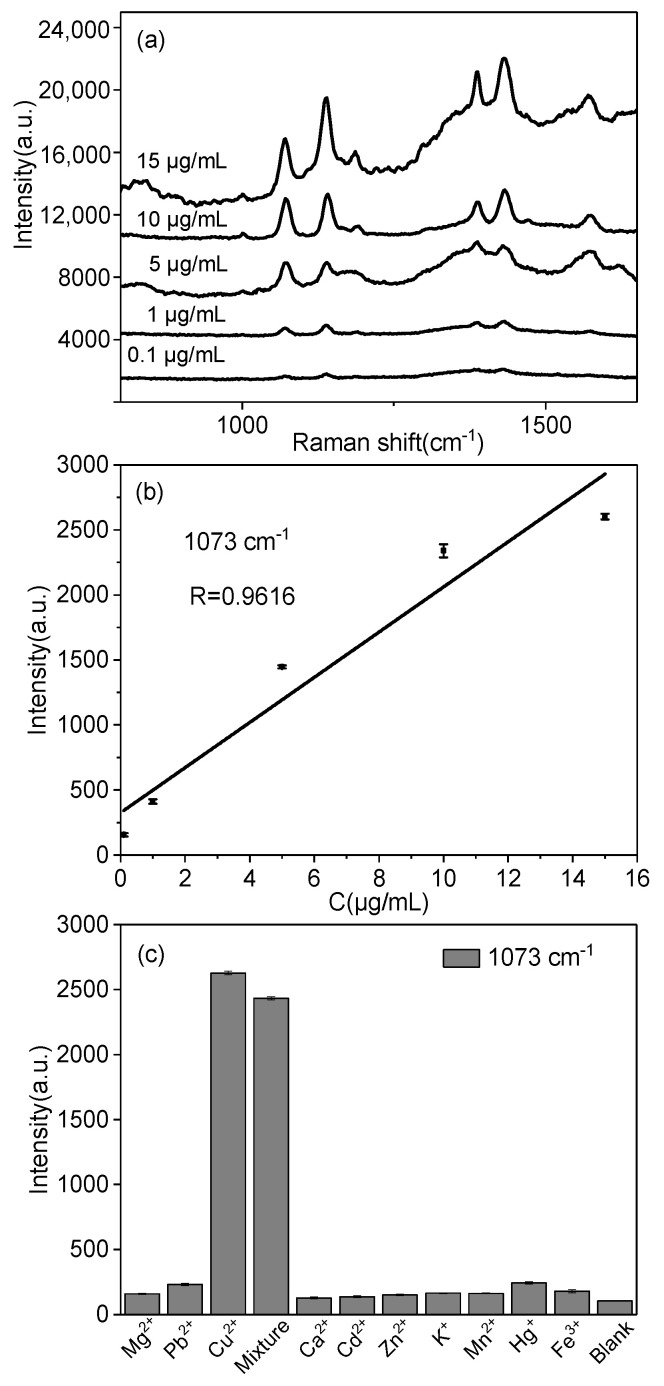
SERS spectra of L−cysteine−functionalized wafers with L−cysteine−modified Ag@SiO_2_/Au tags (**a**) at different concentrations of Cu^2+^, (**b**) SERS peak intensity at 1073 cm^−1^ as a function of Cu^2+^ concentration and (**c**) the histogram of the SERS intensity as different metallic ions presented in the solution.

The Ag@SiO_2_/Au composite was used for detecting Cu^2+^ at different concentrations (15, 10, 5, 1, and 0.1 μg/mL), and the SERS spectra are shown in Figure 5a. The obvious SERS signal of the Ag@SiO_2_/Au tag was observed even with the concentration of Cu^2+^ down to 0.1 ug/mL. As shown in Figure 5b, the intensity of the Raman peak at 1073 cm^−1^ has a correlation with the concentrations of Cu^2+^ between 0.1 ug/mL and 15 ug/mL, in which the relative standard deviation (R^2^) is 0.9616. Control experiments were developed for assessing the selectivity of different metallic ions by this method. Several common ions (Pb^2+^, Mg^2+^, Ca^2+^, Zn^2+^, Cd^2+^, K^+^, Mn^2+^, Hg^+^, and Fe^3+^) were selected as interference ions in water. Figure 5c shows the corresponding histogram of the SERS intensity variation. The results demonstrated that the SERS intensities of the interference metallic ions decreased obviously at 1073 cm^−1^, whereas for Cu^2+^, intense SERS intensity presented at 1073 cm^−1^. However, for the mixture containing Cu^2+^, its SERS intensity was significantly different from most other cations, which is almost the same as that of a single Cu^2+^ solution. Those results demonstrate the excellent selectivity of the developed sensor for Cu^2+^ detection.

## 3. Experimental Section

### 3.1. Materials and Chemicals

Trisodium citrate (Na_3_C_6_H_5_O_7_), silver nitrate (AgNO_3_), Chloroauric acid (HAuCl_4_·3 H_2_O), P-Aminothiopheno (PATP), tetraethyl orthosilicate (TEOS), 3-aminopropyltrimethoxysilane (APTMS), and ethanol were produced by Innochem (Beijing, China). L-cysteine, copper (II) nitrate, magnesium chloride, lead chloride, calcium chloride, zinc nitrate, potassium nitrate, cadmium chloride, manganese sulfate, mercuric chloride, ferric chloride were all bought from Sigma-Aldrich (Shanghai, China). Ammonia hydroxide (NH_3_·H_2_O) was supplied from Shenyang Tiangang chemical reagent factory (Shenyang, China). The reagents used in the experiment are all of analytical grade.

### 3.2. Synthesis of Ag Nanoparticles and Au Nanoparticles

Ag colloids were synthetized by the method of trisodium citrate reduction of Lee’s method [44] and slightly modified. A total of 200 mL of AgNO_3_ solution (1 mM) was heated to reflux with stirring; then, 4 mL 1% trisodium citrate aqueous solution was quickly added to the boiling liquid; finally, the yellow–green solution was obtained after continuous stirring for 40 min, and the mixture was naturally cooled to room temperature. Then, the synthesized Ag NPs colloid was about 60 nm in size.

According to the method of Natan’s team [45], Au colloids 20 nm in size were produced. A total of 100 mL of 1 mM HAuCl_4_ was heated and stirred simultaneously; 10 mL 1% trisodium citrate was added into the solution rapidly when it was boiling; the color of the solution turned from yellow to black and then to wine red in a few minutes. After a reflux of 30 min, Ag colloids were obtained and cooled at room temperature.

### 3.3. Fabrication of Ag @SiO_2_ SERS Tags

The Ag colloids fabricated above were all centrifuged at 1500 rpm for 5 min in order to remove those particles in the rod shape. The Raman probe molecule, PATP, was used to modify on the surface Ag NPs. Briefly, 5 μL PATP ethanol solution (2 mM) was added into 10 mL Ag colloids with a stir reaction for 30 min and isolated by centrifugation at 6500 rpm for 10 min. Subsequently, the centrifuged aggregate was dispersed in the dispersant with a mixture of ethanol and deionized water (7.5 mL and 2.5 mL, respectively). Then, NH_3_·H_2_O (25–28%) of 0.5 mL was added into the suspension as a catalyst to accelerate the reaction process under the condition of stirring for 5 min, adding 10 μL TEOS into the solution system to obtain a complete SiO_2_ shell, and the reaction was continuously stirred at room temperature for 6 h. Finally, the core–shell structure of Ag@SiO_2_ was obtained; the mixed solution was centrifuged at 5500 rpm for 10 min to remove the supernatant of each suspension.

### 3.4. Functionalization of Ag@SiO_2_ SERS Tags

APTMS was used to graft the amino group on the surface of Ag@SiO_2_ nanoparticles. The synthesized Ag@SiO_2_ nanoparticles were centrifugalized and cleaned three times by ethanol; then, they were dispersed in anhydrous ethanol (10 mL). The solution was continuously stirred after the addition of 10 μL for 6 h at room temperature. Then, the aminated composites were obtained and centrifuged to wash them three times. In addition, Ag@SiO_2_/Au composites were prepared by mixing the aminated Ag@SiO_2_ nanoparticles with Au colloids (*v*/*v* 1:1) at different concentrations. The mixture was kept shaking for 30 min and centrifugated to remove the unattached Au nanoparticles. Afterwards, various amounts of L-cysteine solution (10, 20, 30, 50 μL) were added in the Ag@SiO_2_/Au system, then stirred for 2 h at room temperature.

### 3.5. Preparation of L-cysteine-Functionalized Wafers and Cu^2+^ Detection

The glass slides were cleaned in ethanol under ultrasonic condition for 5 min and dried at room temperature. Then, they were immersed in 1 wt% APTMS solution for 12 h to establish amino on the surface. Then, Au colloids were used to submerge the glass slides. Next, those wafers were put into the 2 mM solution of L-cysteine and followed by aqueous Cu^2+^ solution in different concentrations (15–0.1 μg/L).

### 3.6. Characterization

The surface morphology and size of Ag and Ag@SiO_2_ nanoparticles were detected using SU8010 field emission scanning electron microscope (SEM, Hitachi, Tokyo, Japan) and a transmission electron microscope JEM-2100FS (TEM, JEOL, Tokyo, Japan). The UV–vis absorption spectra of Au and Ag colloids and Ag@SiO_2_ nanoparticles were collected on a UV 2400 UV–vis absorption spectrophotometer (Sunny Hengping Instrument, Shanghai, China) using a quartz cell of a 1 cm optical path.

### 3.7. SERS Detection

Briefly, 2 mM PATP was selected as the internal Raman standard molecule, and the SERS performance of Ag@SiO_2_ nanoparticles was evaluated. SERS detection was achieved by laser irradation on the wafers soaked in the Ag@SiO_2_/Au colloid. A portable Raman spectrometer (BWS465 iRman; B&W Tek, Plainsboro, NJ, USA) was used to collect Raman spectra. The excitation wavelength was 785 nm with a spot diameter at 105 µm. In this experiment, a 1.5 m bifurcated fiber probe was chosen to irradiate the wafers so as to obtain the SERS signal, where the laser power and the acquisition time were adjusted to 20 mW and 3 sec, respectively. All Raman data were processed by BWSPEC 4.10 software, collected in the range of 300~3000 cm^−1^.

## 4. Conclusions

In conclusion, we proposed a facile and simple strategy for the preparation of Ag@SiO_2_/Au SERS tags via a mixed solution of water and ethanol. The synthesized core–shell Ag@SiO_2_/Au SERS tags showed excellent stability in high-salt solution and can be preserved over a long period of time. The Ag@SiO_2_/Au SERS tag also provided an intense SERS signal of the embedded Raman probe molecule that enables sensitively and quantitative detection of copper ions in the water environment by SERS. The Ag@SiO_2_/Au SERS tags were functionalized with L-cysteine by the sulfydryl group. The aminoacetic group has a strong coordinate interaction with copper ions. The SERS performance for detecting Cu^2+^ was optimized, and the Ag@SiO_2_ SERS sensor was successfully used for monitoring Cu^2+^ in water with high sensitivity (0.1 ppm) and excellent selectivity. This study demonstrated that the prepared Ag@SiO_2_/Au SERS tags could be used for detection of other ions, pollutants, or biomolecules if corresponding specific interaction is employed, and the tags have potential application in environmental monitoring and immunoassay analyzing.

## Figures and Tables

**Table 1:** Detection of Cu^2+^ in water media based on different SERS substrates and Raman reporters.

Method	LOD	Liner Range	System	Probers	References
SERS	0.1 μM	-	Au NR-PCL	TMT	[31]
SERS	5 μg/L	10 μg/L–1000 mg/L	Au–Pt nanozyme	TMB	[32]
Fluorescent	0.551 nM	0.11–58.0 µg/L	PQ-IIP µPADs	QDs	[33]
Fluorescent	0.3 mM	1 mM–25 mM	aminophenylboronic acid	B,N-carbon dots	[34]
Colorimetry	0.1 μM	0.1 μM–10 μM	AgNPs	-	[35]
This work	0.1 μg/mL		Ag@SiO_2_	PATP	-

## Data Availability

All data presented are available in this article and its Supplementary Materials.

## Supplementary Materials

The following supporting information can be downloaded at: https://www.mdpi.com/article/10.3390/molecules29071503/s1, Figure S1: UV-vis spectra of Ag@SiO_2_ and Ag@SiO_2_/Au composite assembled with different concentrations of Au colloid.
