# Supplementary material for: Fabrication and Application of Ag@SiO2/Au Core–Shell SERS Composite in Detecting Cu2+ in Water Environment
Source: Molecules. 2024 Mar 27;29(7):1503. doi: 10.3390/molecules29071503 (PMC11013303; doi:10.3390/molecules29071503)
Supplement: Supplementary file 1 [file molecules-29-01503-s001.zip › molecules-2922792-supplementary.pdf]

Fabrication and application of Ag@SiO_2/Au core-shell SERS composite in detecting Cu^{2+} in water environment

Meizhen Zhang [a], Lin Meng[a,c], Kelgenbaev Kalyinur[c], Siyuan Dong[a,c], Chang Xinyi [a], Qian Yu [a], Rui Wang[a*], Bo Pang [b*], Xianming Kong [a]

[a] School of Petrochemical Engineering, Liaoning Petrochemical University, Fushun, Liaoning 113001, P. R. China

[b] Department of Materials and Environmental Chemistry, Stockholm University, 10691 Stockholm, Sweden

[c] International Education College, Liaoning Petrochemical University, Fushun, Liaoning 113001, P. R. China

*Corresponding author: rwang@lnpu.edu.cn; bo.pang@mmk.su.se

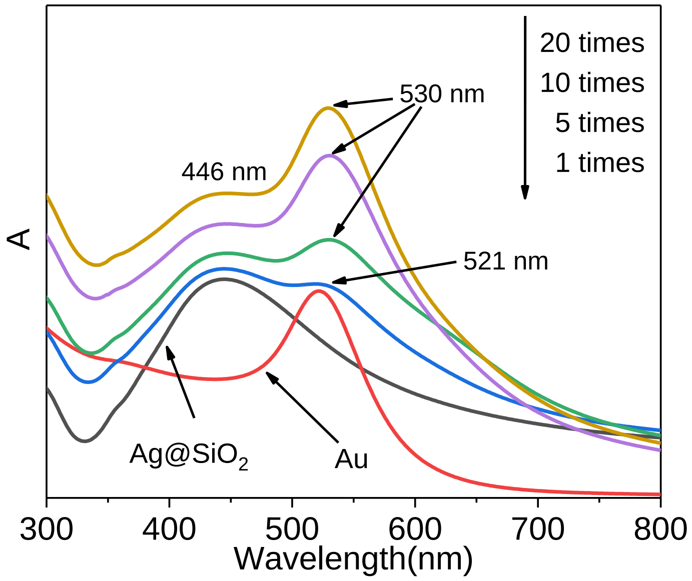

Figure S1. UV-vis spectra of Ag@SiO_2 and Ag@SiO_2/Au composite assembled with different concentrations of Au colloid
